# 2, 6-dichlorobenzonitrile Causes Multiple Effects on Pollen Tube Growth beyond Altering Cellulose Synthesis in *Pinus bungeana* Zucc

**DOI:** 10.1371/journal.pone.0076660

**Published:** 2013-10-11

**Authors:** Huaiqing Hao, Tong Chen, Lusheng Fan, Ruili Li, Xiaohua Wang

**Affiliations:** Key Laboratory of Plant Molecular Physiology, Institute of Botany, Chinese Academy of Sciences, Beijing, China; Iowa State University, United States of America

## Abstract

Cellulose is an important component of cell wall, yet its location and function in pollen tubes remain speculative. In this paper, we studied the role of cellulose synthesis in pollen tube elongation in *Pinus bungeana* Zucc. by using the specific inhibitor, 2, 6-dichlorobenzonitrile (DCB). In the presence of DCB, the growth rate and morphology of pollen tubes were distinctly changed. The organization of cytoskeleton and vesicle trafficking were also disturbed. Ultrastructure of pollen tubes treated with DCB was characterized by the loose tube wall and damaged organelles. DCB treatment induced distinct changes in tube wall components. Fluorescence labeling results showed that callose, and acidic pectin accumulated in the tip regions, whereas there was less cellulose when treated with DCB. These results were confirmed by FTIR microspectroscopic analysis. In summary, our findings showed that inhibition of cellulose synthesis by DCB affected the organization of cytoskeleton and vesicle trafficking in pollen tubes, and induced changes in the tube wall chemical composition in a dose-dependent manner. These results confirm that cellulose is involved in the establishment of growth direction of pollen tubes, and plays important role in the cell wall construction during pollen tube development despite its lower quantity.

## Introduction

Pollen tubes are cells that grow from their tips. Their elongation exhibits a polarized pattern of growth similar to that of fungal hyphae, root hairs and neuronal axon guidance in animals [1], [2]. Pollen tubes grow long distances and transport male gametes toward the embryo sac, in which fertilization occurs. During the process of pollen tube growth, secretory vesicles derived from the Golgi apparatus transport large amounts of membrane and cell wall precursors to the tip [3], [4]. At the apex, secretory vesicle membranes fuse with the plasma membrane, releasing the precursors and forming a new cell wall at the pollen tube tip, and then pollen tube elongates [5], [6], [7]. It is generally accepted that the wall of the pollen tube differs from that of other plant cells [8], [9], [10], and although the precise composition and structure of the pollen tube wall remain uncertain, several differences between angiosperm and gymnosperm pollen tube walls have been identified [11], [12], [13]. In angiosperms, the pollen tube wall contains two layers: an outer layer of mainly cellulose and pectin and an inner callosic layer [14], [15], [16]. In contrast, the pollen tube wall in gymnosperms also consists of cellulose, pectin and callose, however, there was no separate callosic layer in gymnosperms [17]. In recent years, the deposition and construction of angiosperm pollen tube wall have been extensively studied, however, our knowledge of the pollen tube wall of gymnosperms remain limited [17], [18].

In vegetative cells, cellulose is the most abundant polysaccharide and provides the framework for the wall [19]. In contrast, the cellulose content is very low in pollen tubes, comprising only 2–10% of the tube wall [20]. Cellulose has been found in the pollen tube walls of petunia [21] and *Nicotiana alata* [22]. Using cellobiohydrolase I (CBHI) probes, Ferguson *et al.* [11] identified cellulose in the inner layer of the pollen tube wall of *Nicotiana tabacum*. In the *Pinus sylvestris* pollen tube, cellulose microfibrils were found in the pollen tube wall [17]. It has been suggested that the cellulose synthase complex (CSC) at the plasma membrane is responsible for cellulose synthesis [23], [24], [25], [26]. Despite numerous chemical and physical studies of cellulose in the tube wall, the precise function of cellulose in pollen tube, especially in gymnosperms is still a matter of conjecture due to the lack of suitable biological materials and techniques.

The compound 2, 6-dichlorobenzonitrile (DCB) is an effective and specific inhibitor of cellulose synthesis in higher plants [27]. It inhibits the polymerization of Glc into β-1, 4-linked glucan and affects β-1, 4-glucan crystallization at the plasma membrane [28], [29]. In recent years, DCB has been used to study cellulose synthesis process in various cells, including tobacco, bean cell suspensions, tomato and *Arabidopsis* [29], [30], [31]. Anderson *et al.* [32] showed that DCB treatment affected pollen tube morphology, leading to the rupture of the tube and the deposition of callose in lily and petunia pollen tubes, respectively. DCB also inhibited the mobility of YFP-CESA6 complexes at the plasma membrane and caused the hyperaccumulation of CESA [33]. In our previous study, we found that DCB affected the seedling growth and blocked the dynamics of Golgi and TGN [34]. However, the effects of DCB on gymnosperm pollen tube growth had not been extensively studied. The objective of this investigation was to explore the effect of DCB on *Pinus bungeana* pollen germination and pollen tube growth. The relationship between the inhibition of cellulose synthesis and changes in the chemical composition and the ultrastructure of pollen tube wall was analyzed in detail.

## Results

### Effects of DCB on Pollen Germination and Pollen Tube Growth

**Figure 1:**
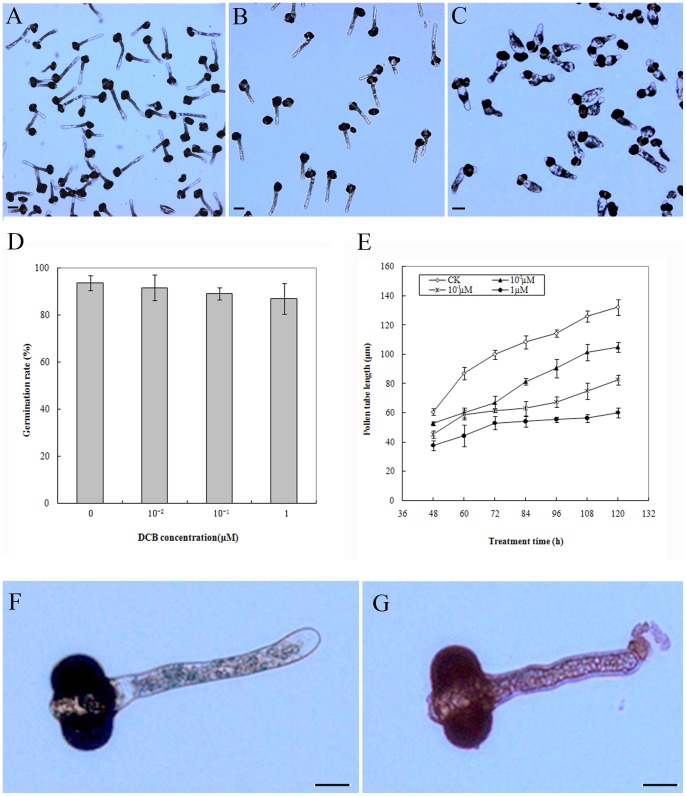
Effects of DCB on pollen germination and pollen tube growth of *Pinus bungeana* Zucc. Bars = 60 µm (A, B, C), 20 µm (F, G). A. The control pollen tube growing in 1% ethanol, showing normal length and shape. B. Pollen tubes cultured in normal medium for 3 d, showing normal shape and long tube. C. Pollen tubes treated with 10^−1 ^µM DCB for 3 d, the tip and /or the base of pollen tube swelled. D. DCB slightly affected germination percentage of pollen grains of *Pinus bungeana.* Only pollen tubes that were longer than the diameter of pollen grain were measured. Mean values are shown with SD of three experiments. E. DCB inhibited the pollen tube growth of *Pinus bungeana.* Only pollen tubes that were longer than the diameter of pollen grain were measured. Mean values are shown with SD of three experiments. F. Pollen tubes cultured in normal medium for 3 d, showing normal shape. G. Pollen tubes cultured in normal medium for 3 d and then treated with DCB for 6 h, showing the tube tip ruptured (arrow).

The following results were obtained from three separate experiments using the same batch of pollen. In normal germination medium, pollen tubes appeared healthy with regular shapes. As shown in Figure 1A and B, control pollen tubes were long, with a constant diameter and a clear zone at the region of the tip. The morphology of pollen tubes treated with 10^−2 ^µM DCB was very similar to that of control pollen tubes. In contrast, addition of 10^−1 ^µM DCB to the medium at the beginning of the culture period caused morphological abnormalities in pollen tubes: the tip or/and base of the tube swelled, the diameter of tube increased, and the clear zone at the tip disappeared in most of the pollen tubes (Figure 1C). Increased numbers of abnormal pollen tubes appeared at greater DCB concentrations. Almost all of the pollen tubes grown in the presence of 1 µM DCB appeared swollen and rounded. However, DCB had a slight effect on the germination percentage of pollen grains (Figure 1D). In the presence of DCB, the germination percentage was slightly lower than the 93.7% germination of control pollen grains, but there were no significant differences in the germination percentage in pollen grains grown with different DCB concentrations. The average growth rate of pollen tubes treated with DCB was distinctly slower than that of the control: the average growth rate of control pollen tubes was 24.1 µm/d, whereas the growth rate was only 12.6 µm/d in the presence of 10^−1 ^µM DCB (Figure 1E).

To examine whether DCB affects pollen tubes that have begun to grow normally, pollen grains were allowed to grow in normal germination medium for 3 d, and then DCB was added to the medium to a final concentration of 1 µM. The morphology of the pollen tubes was examined every 6 h. When DCB was added to the medium, the pollen tubes began to rupture at the tip within 6 h (Figure 1F, 1G), and the rupture rate increased with increasing treatment times (Figure S1). Normally growing pollen tubes of other growth stages were also examined. The results showed that the addition of DCB caused pollen tubes to rupture at the tip irrespective of the length of the growth period in normal medium.

### DCB Affects the Organization of Cytoskeleton of *Pinus bungeana* Pollen Tube

**Figure 2:**
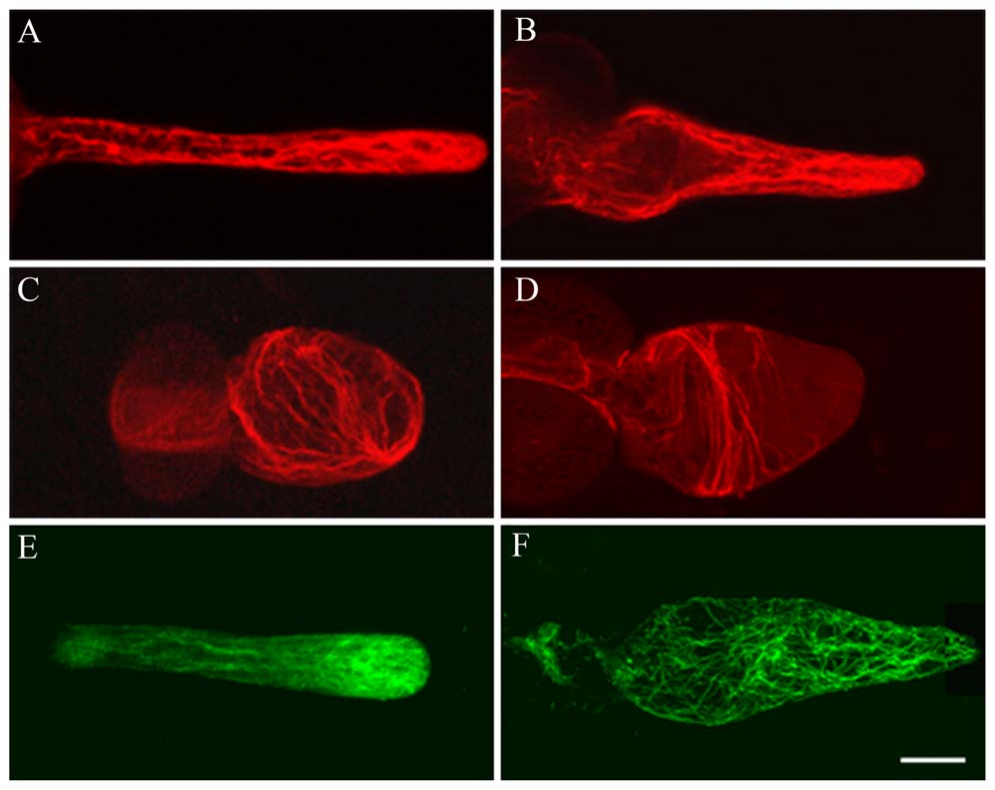
Effects of DCB on the reorganization of the cytoskeleton in pollen tubes. Bar = 100 µm. A. Control pollen tubes, showing long pollen tube and normal organization of actin filaments distributed throughout the tube. B. Pollen tubes treated with 10^−1^ µM DCB, showing actin filaments at the basal part of the tube were destroyed, while the organization of actin filaments at the tip was not affected. C. Pollen tubes treated with 1 µM DCB for 3 d, showing the swollen pollen tubes and the reorganization of actin filaments. D. Pollen tubes treated with 1 µM DCB for 4 d, showing actin filaments were reorganized and mainly vertical to the direction of elongation of the pollen tube. E. In the control pollen tube, microtubules were distributed throughout the pollen tube in a net axial array, mainly parallel to the direction of elongation. F. Pollen tubes treated with 10^−1^ µM DCB for 4 d, microtubule arrangement was disrupted.

Actin filaments have been reported to be involved in the vesicle trafficking, delivery of CESA and the cell wall construction [34], [35], [36]. In addition, actin filaments play an important role in the tube tip growth, thus we compared the actin cytoskeleton in control and DCB-treated pollen tubes. We found that actin filaments in control tubes were organized into a network of bundles throughout the length of pollen tube except at the tip (Figure 2A). However, DCB affected the organization of actin filaments in a dose-dependent manner. Low concentration of DCB induced the slight fragmentation in actin filaments, especially in the swollen region of the tube (Figure 2B). High concentration markedly disturbed the array and organization of actin cytoskeleton, actin filaments were mainly vertical to the growth axis and there was no actin filament at the tube tip (Figure 2C, 2D). We also examined the effects of DCB on the microtubule (MT) cytoskeleton. MT has been proved to be involved in the movement of cellulose synthesis enzyme. We found that when immunolabelled with the anti-β-tubulin antibody, MTs also formed a continuous network throughout the pollen tube except the tube tip, where MTs were enriched but distributed in a radial array (Figure 2E). However, DCB treatment led to the obvious fragmentation and disruption of MTs in the tube (Figure 2F).

### FM4-64 Internalization is Disrupted by DCB Treatment

**Figure 3:**
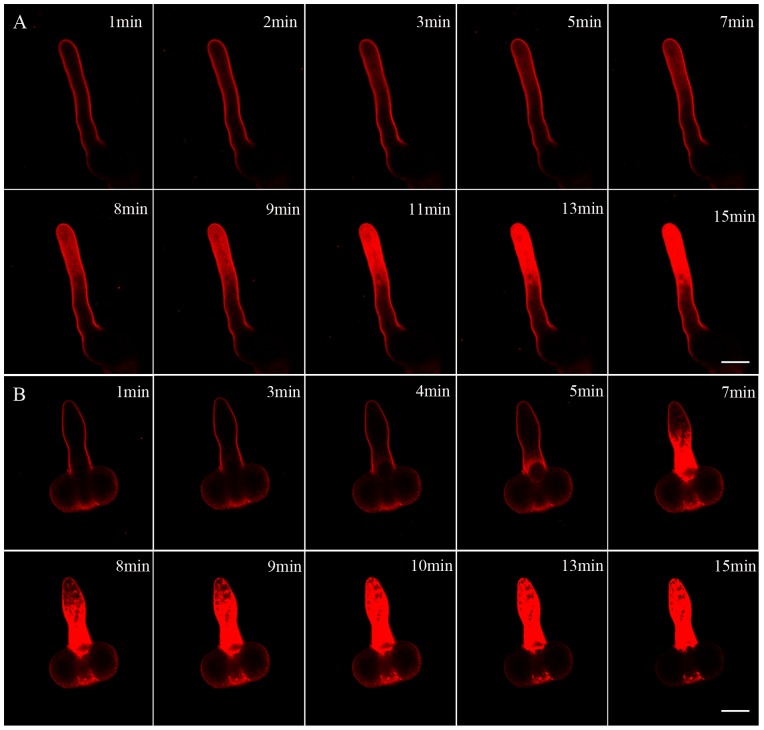
Effects of DCB on the FM4-64-uptake time course in growing *Pinus bungeana* pollen tubes. Bar = 25 µm. A. Confocal fluorescence images of FM4-64 internalization in the control pollen tubes at different times, the rapid uptake suggests a high rate of endocytosis and membrane trafficking in the pollen tube. B. In the DCB-treated pollen tubes, the uptake of FM4-64 occurred at the basal part of tube and the internalization process was activated by DCB treatment.

As DCB affects the organization of cytoskeleton and FM4-64 can be used as reliable markers of membrane trafficking events in plant cells [37], [38], we analyzed the effects of DCB on the FM 4-64 internalization of pollen tube. In the control pollen tube, the internalization of FM4-64 into the tube followed a strict time sequence and showed distinct time-dependent internalization. After staining with FM 4-64 for 1 min, the fluorescence was observed at the plasma membrane. As the increase of incubation time, strong fluorescence of FM 4-64 was apparent and finally a typical staining pattern was formed after 15 min following the dye application (Figure 3A). After treatment with DCB, FM4-64 staining was mainly observed at the basal part of the pollen tube. In addition, the internalization process of FM4-64 was greatly accelerated, taking approximately 10 min to reach saturation (Figure 3B).

### Ultrastructural Changes in the Tube Wall and Organelles of the Pollen Tube

**Figure 4:**
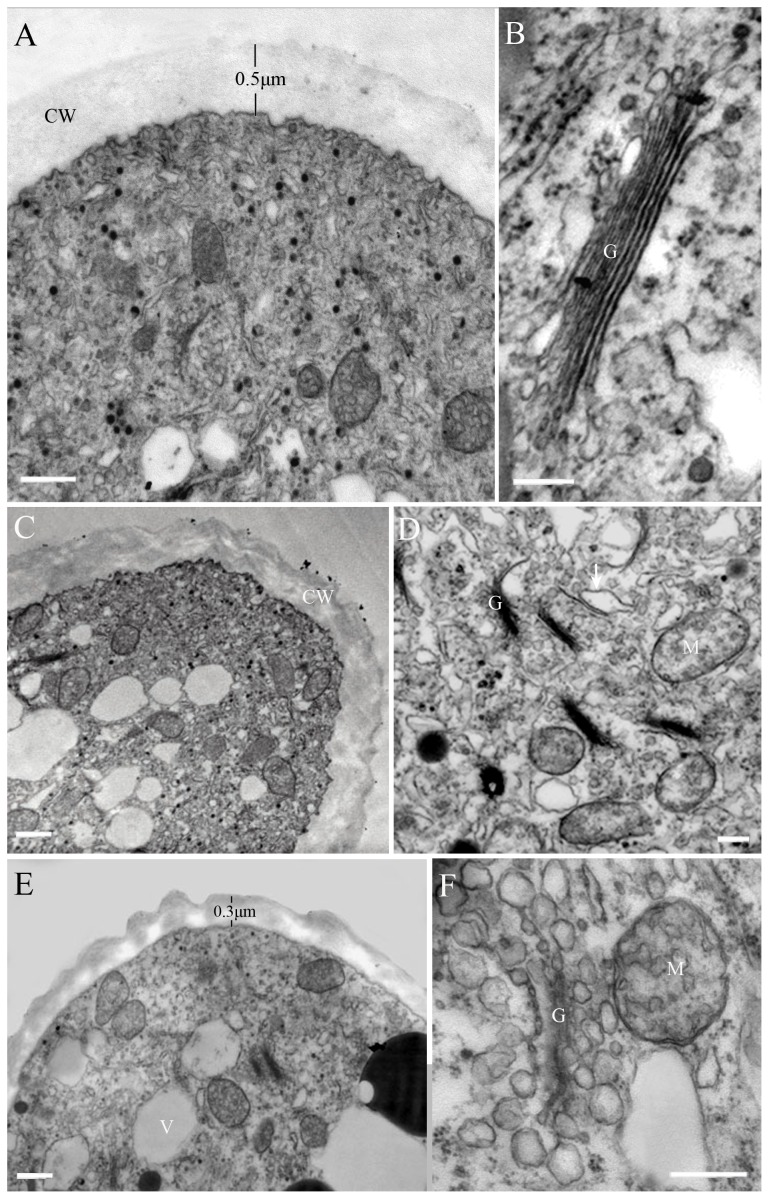
Effects of DCB on the ultrastructure of pollen tube of *Pinus bungeana* Zucc. Bars = 1 µm (A, C, E), 0.2 µm (B, D, F). CW, cell wall; G, Golgi; M, mitochondria; V, vacuole. A. Tip region of a control normal pollen tube, showing the vesicles and organelles at the apical clear zone. The cell wall was uniform and 0.5 µm in thickness. B. Normal pollen tube tip, showing typical morphology of Golgi and endoplamic reticulum. Golgi contains 6 cisterna and shows a distinct cis-to-trans polarity. C. Pollen tubes treated with 10^−1^ µM DCB for 3 d, showing the loosen tube wall and the vacuoles at the tip. D. Pollen tubes treated with 10^−1^ µM DCB for 4 d, the cisternae of Golgi and the membrane of mitochondria began to disrupt (arrow). E. Pollen tubes treated with 1 µM DCB for 3 d, showing the thin tube wall and numerous vacuoles in the pollen tube tip. The cell wall became thinner compared to the control and was about 0.3 µm in thickness. F. Pollen tubes treated with 1 µM DCB for 3 d, showing the disruption of Golgi near the vacuoles.

Ultrastructural analysis showed that the greatest differences between the untreated and DCB-treated pollen tubes were in the tube tips. In untreated pollen tubes, the uniform tube wall was about 0.5 µm thick. The pollen tube tip was filled with a dense cytoplasm enriched with a large amount of organelles, including endoplasmic reticulum, Golgi, and mitochondria. Various types of vesicles were present (Figure 4A) and most Golgi consisted of six to eight flattened cisternae surrounded by numerous secretory vesicles (Figure 4B). We found that DCB affected the tube wall in a dose-dependent manner. In the low concentration of DCB (10^−1^ µM), a more loosely packed tube wall was observed and some vacuoles were found (Figure 4C). While 1 µM DCB induced a distinct decrease in the thickness of the tube wall (0.3 µm) and the accumulation of vacuole was observed in the tube tip (Figure 4E). The ultrastructural investigations also showed that DCB damaged the ultrastructure of the major organelles. In pollen tubes treated with 10^−1^ µM DCB, the number of secretory vesicles in the pollen tube tip was lower than that in the control, cisternae of Golgi became disrupted, and mitochondria membranes ruptured (Figure 4D). At 1 µM DCB, most of the cytoplasm in the tip region had disappeared, the Golgi was disaggregated (Figure 4F).

### Changes in the Localization of Cellulose, Callose and Pectins after DCB Treatment

**Figure 5:**
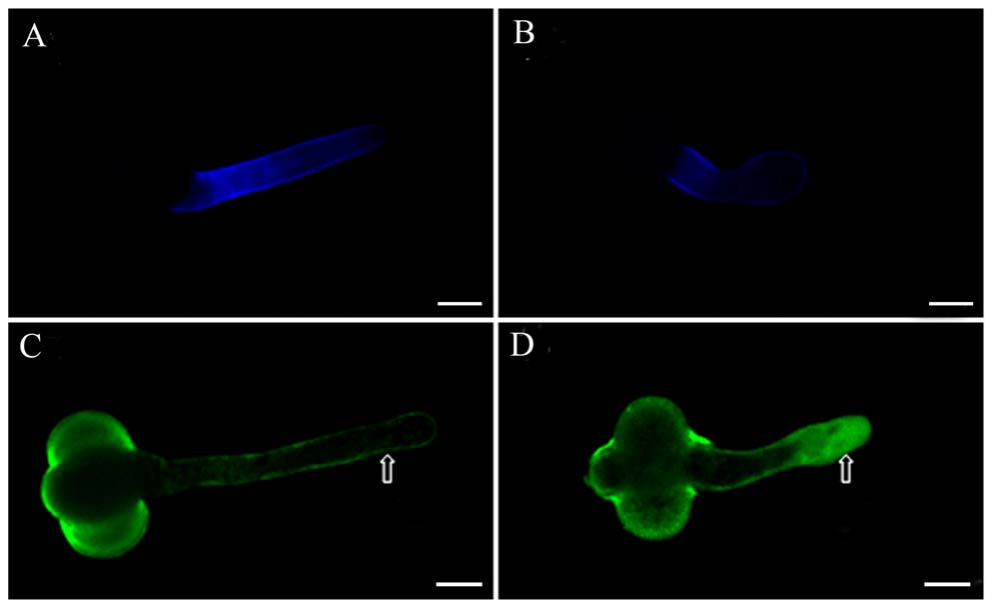
Effects of DCB on the distribution of cellulose and callose in tube walls of *Pinus bungeana* Zucc. Bars = 20 µm A. Calcofluor stained control pollen tubes, showing cellulose was distributed along the whole length of pollen tube. B. Calcofluor stained DCB-treated pollen tubes, showing less cellulose was present at the pollen tube wall, especially the tube tip. C. Pollen tubes cultured in normal medium for 3 d, showing strong fluorescence, excited from callose on the whole surface of the pollen tube. D. Pollen tubes treated with DCB for 3 d, showing strong fluorescence at the pollen tube tip.

We labeled the cellulose in the tube wall by using calcofluor. The results showed that in the control pollen tube, fluorescence was visible throughout the pollen tube wall, including the elongation tube tip. It was important to note that the fluorescence intensity at the tube tip was significantly lower than that in the subapical region, indicating that cellulose content was low in the tube tip (Figure 5A). In the DCB-treated pollen tubes, the distribution of cellulose was still present throughout the tube wall, but the fluorescence intensity declined significantly (Figure 5B), and the fluorescence was difficult to distinguish in the swelled region of the tube wall. To further analyze the change of cellulose content quantitatively, we isolated the cell wall material of pollen tube and determined the cellulose content. We found that DCB treatment deduced the cellulose content in the tube wall in a dose manner. In the presence of 1 µM DCB, pollen tubes with a reduction of 52%, showed a much lower amount of cellulose content as compared to the control (Table 1). The distribution of callose in pollen tubes was examined by laser scanning confocal microscopy after staining with aniline blue. In pollen tubes incubated in normal medium, callose fluorescence was observed along the entire surface of the pollen tube wall, with no distinct difference in the fluorescence intensity between the tip and other regions of the pollen tube (Figure 5C). However, in the presence of DCB, strong fluorescence was observed at tips of pollen tubes (Figure 5D).

**Table 1:** The effects of DCB on cellulose content in the pollen tube of *Pinus bungeana* Zucc.

	Cellulose of total CWM
Control	3.98±1.13
10^−1^ µM DCB	2.86±1.25
1 µM DCB	1.92±0.90

**Figure 6:**
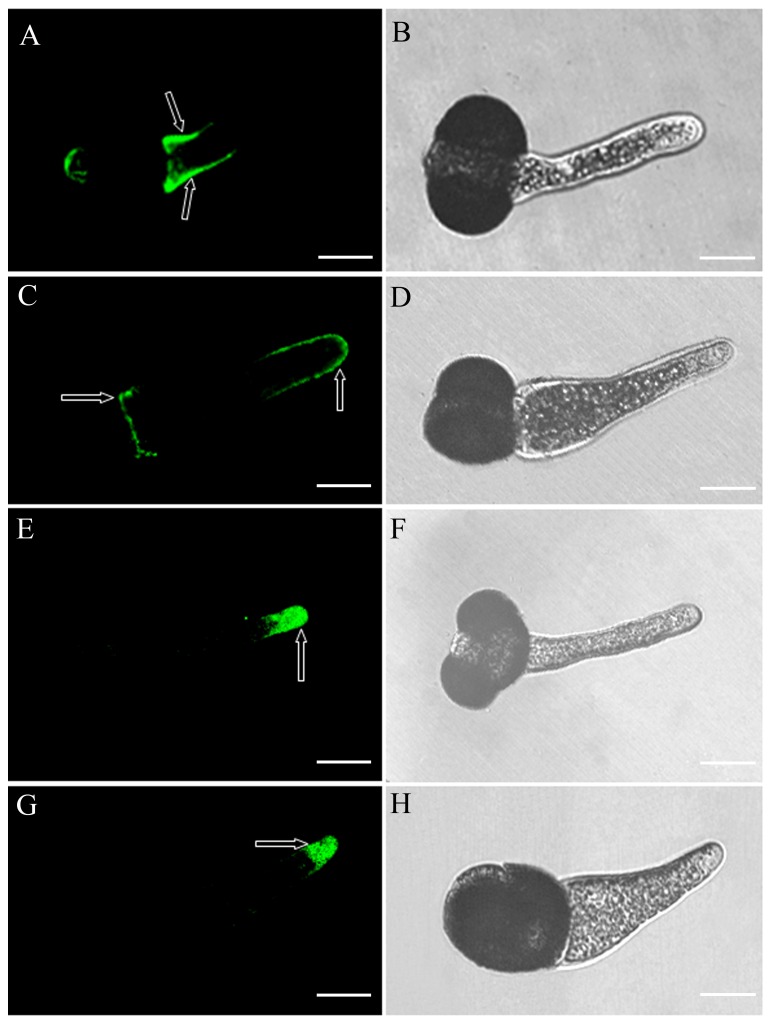
Fluorescence after immunolabeling of *Pinus bungeana* Zucc. pollen tubes with JIM5 and JIM7. Bars = 20 µm A. Fluorescence after antibody JIM5 labeling of pollen tubes cultured for 3 d in normal medium (indicated by arrows). B. Corresponding bright-field image showing strong fluorescence occurred at the germination site and the basal part of pollen tube wall. C. Fluorescence was observed mainly at the tube tip after antibody JIM5 labeling of pollen tubes cultured for 3 d in the presence of 10^−1^ µM DCB (indicated by arrows), D. Corresponding bright-field image showing that faint fluorescence occurred at the germination site and pollen tube tip. E. Fluorescence after antibody JIM7 labeling of pollen tubes cultured for 3 d in normal medium (indicated by arrows). F. Corresponding bright-field image showing that fluorescence occurred at the pollen tube tip. G. Fluorescence after antibody JIM7 labeling of pollen tubes cultured for 3 d in the presence of 10^−1^ µM DCB (indicated by arrows). H. Corresponding bright-field image showing that fluorescence occurred at the pollen tube tip.

We further investigated the changes of pectin distribution in the pollen tube wall by using JIM5 JIM7, which recognize the acidic pectin and esterified pectin, respectively. In control pollen tubes, strong fluorescence from JIM5 labeling was observed at the germination site (Figure 6A, 6B), and JIM7 labeled the pollen tube tip (Figure 6E, 6F). In contrast, in pollen tubes treated with DCB, faint fluorescence from JIM5 labeling was visible at the germination site and the front of the tube wall including the tip (Figure 6C, 6D), whereas fluorescence representing JIM7 labeling was present at the pollen tube tip in a fluorescence pattern similar to that of control tubes (Figure 6G, 6H).

### FTIR Analysis of the Pollen Tube Wall

**Figure 7:**
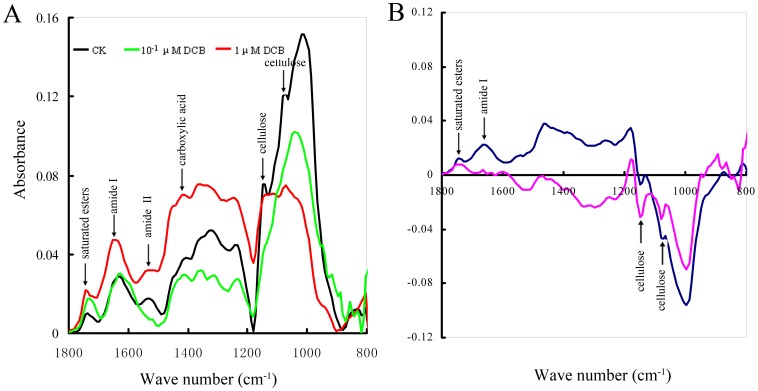
Fourier transform infrared (FTIR) spectrum obtained from the apical region of *Pinus bungeana* pollen tubes cultured for 3 d. A. The FTIR spectra obtained from the tip region of the control pollen tubes and that of treated with different concentrations of DCB revealed that DCB induced changes in absorbance and /or the displacements of the peaks. CK: the control pollen tube. B. The difference spectra generated by digital subtraction of the spectra of control tube walls from the spectra of DCB-treated tube walls, showing that the content of proteins and esterified pectins increased, while the cellulose content decreased.

To further investigate the effects of DCB on pollen tube growth, the chemical composition of the tube wall was further analyzed by using FTIR. Figure 7A shows that FTIR spectra obtained from control pollen tubes were distinctly different from those of DCB-treated pollen tubes. Control pollen tubes (CK) were grown in normal germination medium for 3 days, and then were analyzed by FTIR. The results showed that saturated esters absorbed at 1743 cm^−1^, carboxylic acid at 1411 cm^−1^, amide-stretching bands of protein at 1643 cm^−1^ and 1535 cm^−1^, carbohydrates between 1200 cm^−1^ and 900 cm^−1^, and cellulose peaks at around 1149 cm^−1^ and 1072 cm^−1^. The FTIR spectrum of pollen tubes in the presence of DCB changed in absorbance intensity and the locations of specific peaks. In the presence of 10^−1^ µM DCB for 3 days, the saturated esters absorbed at 1735 cm^−1^, carboxylic acid at 1419 cm^−1^, and amide-stretching bands of protein at 1635 cm^−1^. In the presence of 1 µM DCB, the saturated esters absorbed at 1743 cm^−1^, carboxylic acid at 1434 cm^−1^, and amide I at 1651 cm^−1^. The difference spectrum generated by digital subtraction of the CK spectra from that of DCB (Figure 7B) revealed that the saturated ester peak and the amide stretches increased, while cellulose content distinctly decreased with increasing of DCB concentration.

## Discussion

Pollen tubes exhibit many characteristics that are different from those of other plant cells. Most notably, pollen tubes elongate by tip growth, whereas many other cells expand by diffuse growth, and the pollen tube attains its tubular shape *via* a polarized tip-growth mechanism [7], [39], [40]. In the present study, pollen tubes cultured in normal germination medium had a normal shape and length. When DCB was added to the medium at the start of the culture period, the pollen grains were able to germinate at all DCB concentrations examined, with no significant differences in germination percentages among the DCB concentrations tested. However, the morphology of the pollen tubes varied in the presence of different DCB concentrations. At 10^−2^ µM DCB, no morphological changes were apparent, but in the presence of 10^−1^ µM DCB, the pollen tubes appeared swollen at the tip or/and base. With increased DCB concentrations, the rate of abnormalities in the pollen tubes increased, and the average pollen tube growth rate decreased. We speculate that the pollen tube wall is not able to be constructed normally in the presence of DCB, which further affects the properties of the tube wall, leading to swelling. However, we did not observe ruptured pollen tubes at any DCB concentration, as opposed to the rupture events reported in other pollen tubes [33]. This is possibly due to the difference in the construction of angiosperm and gymnosperm pollen tube walls. Derksen *et al.* [17] showed that the cellulose content was higher in gymnosperm pollen tubes than that in angiosperm pollen tubes. Therefore, angiosperm pollen tubes are likely more sensitive to DCB. Another possibility is that the rapid growth rate of angiosperm pollen tubes creates a tendency toward rupture in the presence of DCB. Furthermore, when DCB was added to normally growing pollen tubes, they tended to rupture at the very tip and the cytoplasm flowed into the medium, indicating that cellulose is essential for the establishment of the pollen tube even though it is present in low amounts in the tube wall. The results presented here confirm that there are differences in the aspect of chemical composition between the walls at the tip and at the rear of growing pollen tubes [7], [17], [41].

Cytoskeleton plays important roles in the elongation of pollen tube. In our study, we found that both of the microtubules and actin filaments were disturbed when treated with DCB, indicating that there is a close relationship between the cytoskeleton and cellulose synthesis during the elongation of pollen tube. Microtubules are known to be involved in the transport of CESA complexes from Golgi bodies to the plasma membrane [42] and the specific inhibition of cellulose synthesis by DCB can disorganize the microtubules array. Actin filaments are critical for the cytoplasmic streaming, the vesicle trafficking, and transport the CESA through Golgi bodies [36]. In *Arabidopsis*, we found that DCB inhibited the movement of Golgi bodies and TGN [34], which are essential for the delivery of CESA. Sampathkumar *et al*. [43] found that actin organization affects CesA tracking behavior at the plasma membrane. Thus we conclude that DCB can disrupt the intracellular trafficking in the pollen tube by interfering the actin filaments. To further investigate the effects of DCB on the vesicle trafficking, we labeled the pollen tubes with the endocytic marker, FM4-64. We found that FM4-64 internalization was dependent on the incubation time and finally formed a typical reverse V pattern in the cytoplasm of the pollen tube, which is consistent with the previous study of *Picea meyeri* [44]. In the DCB-treated pollen tubes, the typical reverse V pattern of FM4-64 disappeared and a distinct different distribution of FM4-64 was observed, indicating that the vesicle trafficking was perturbed by DCB. It is important to note that the internalization of FM4-64 was accelerated, indicating that there might be some distinct changes in the composition of cell wall or the mode of endocytosis.

Ultrastructural observations of untreated and DCB-treated pollen tubes showed distinct morphological differences at the pollen tube tip. In the control pollen tube, the tube wall was uniform; the main organelles and secretory vesicles were observed at the pollen tube tip. In the presence of DCB, the loosened and thin pollen tube wall was observed at the very tube tip, which is likely caused by the abnormal deposition of wall material [45]. In addition, in DCB-treated pollen tubes, secretory vesicles disappeared from the tube tip, while the number of vacuoles and mitochondria increased in the apical region. At higher DCB concentrations, the organelles were disorganized and the pollen tube tip tended to be vacuolated. During the pollen tube tip growth, secretory vesicles derived from the Golgi apparatus transport the cell wall materials such as hemicelluloses and pectins needed for cell wall expansion, thus the tip growth requires the integrity of the secretory system in pollen tubes [46], [47]. In the present study, DCB treatment retarded the tip growth by interfering the cytoskeleton and vesicle trafficking in the pollen tube, and thus the structure of the tube wall was changed.

Disruption of cellulose synthesis by DCB affects the morphology of pollen tube, thus we are curious as to whether the chemical composition of tube wall was affected. Calcofluor labeling showed that cellulose was present throughout the control pollen tube wall, including the tube tip. This result differs from that of angiosperm, in which cellulose are absent at the tube tip [11]. Our results confirmed that the density of cellulose in the tube tip was much lower than that in the tube of *P. bungeana* as has been previously reported from other species [17]. However, in the presence of DCB, the fluorescence intensity declined, indicating that there was less cellulose in the pollen tube wall. The results confirm that DCB inhibited the synthesis of cellulose in the pollen tube [28]. In addition, callose, a β-1,3-D-glucan with some 1,6-linked branches, is a major polysaccharide component of the pollen tube wall [11], [14], [48]. Callose can be synthesized in the normal pollen tube walls, unlike in vegetative cell walls [15], [49]. Callose is also present at the tips of growth-inhibited pollen tubes [17], [36], [50]. The results presented here showed that callose was present in the pollen tube walls of *Pinus bungeana.* The pattern of callose deposition was different in control and DCB-treated pollen tubes. In controls, callose fluorescence could be seen along the entire pollen tube, but in the presence of DCB, callose accumulated at the tube tip, suggesting that the growth of the pollen tube was retarded or stopped [50], [51]. Numerous studied reported that both cellulose and callose synthases exist in the plasma membrane and use the same substrate, yet their activities appear to be regulated in an opposing fashion [52], [53]. Therefore, we speculate that DCB is involved in the regulation of synthesis and distribution of callose in the pollen tube.

Pectin is another important polysaccharide component of the pollen tube wall [54]. In the angiosperm pollen tube, pectin is synthesized and modified most actively during elongation [12], [18]. It has been suggested that acidic pectin is derived from the de-esterification of esterified pectin at the tube tip. Our immunolabeling results showed that acidic pectin is mainly distributed at the germination site in the control pollen tube, in accordance with the result of Derksen *et al.* [17]. In DCB-treated pollen tubes, acidic pectin appeared primarily along the front of the tube wall, including the tip. Because acidic pectin can be cross-linked by Ca^2+^, providing mechanical strength to the tip wall [54], the accumulation of acidic pectin at the tube tip might limit the expansion of the tube wall and eventually retard pollen tube growth [7]. Immunolabeling with JIM7 detected esterified pectin at the tips of normal pollen tubes, similar to the findings in *Pinus sylvestris* [17], but there was no change in the distribution of esterified pectin with DCB treatment, leading us to speculate that DCB has little effect on the distribution of esterified pectin in pollen tubes. These results demonstrated that plant cells have the capacity to survive when exposed to DCB, the specific inhibitor that affects cellulose biosynthesis.

FTIR microspectroscopy is a reliable and non-destructive method for analyzing cell wall architecture [55], [56]. Our FTIR microspectroscopic analysis revealed significant changes in the chemical structure of the pollen tube wall in the presence of DCB compared with the structure of control pollen tubes. The difference spectrum demonstrated that the saturated ester and protein content increased, while cellulose content decreased with DCB treatment. This is in accordance with our immunolabeling results. Our data further confirmed that the chemical composition of tube wall changed distinctly when cellulose synthesis was interrupted.

In conclusion, DCB treatment caused the malformation of pollen tubes and retarded the tube growth in *Pinus bungeana.* Inhibition of cellulose synthesis resulted in the changes of chemical components of pollen tube wall. DCB also led to the disruption of the pollen tube wall. We confirm that cellulose plays an important role in tube wall construction during pollen tube elongation despite its low levels in the pollen tube wall. These data also demonstrated that there is a close relationship between the vesicle trafficking, cell wall construction and tip growth of the pollen tube.

## Materials and Methods

### Plant Materials

*Pinus bungeana* cones containing ripe pollen were obtained from the Botanical Garden of the Institute of Botany at the Chinese Academy of Sciences. The collected pollen grains were dried at room temperature overnight and stored at −80°C until use.

### *In vitro* Pollen Germination

*Pinus bungeana* pollen grains were cultured in a germination medium containing 15% sucrose, 0.01% H_3_BO_3_, and 0.01% CaCl_2_. DCB (>98%; Sigma, St. Louis, MO, USA) was dissolved in ethanol and added to the germination medium to final concentrations ranging from 10^−2^ µM to 1 µM. As a control, pollen was also cultured in the presence of ethanol, which has no effect on pollen growth. The cultures were incubated on a rotary shaker at 27°C in darkness.

### Determination of Pollen Germination and Pollen Tube Growth

The percentage of germinated pollen grains was determined from observations with a Nikon microscope (Tokyo, Japan). Pollen grains were considered to be germinated when the length of the tube was greater than the diameter of the pollen grain. The lengths of 200 randomly chosen pollen tubes were measured at 12-h intervals under a light microscope. All experiments were performed at room temperature.

### Fluorescence Labeling of Cytoskeleton

Pollen tubes were fixed in a freshly prepared solution of 4% paraformaldehyde in 50 mM Pipes buffer (pH 6.9) for 1 h at room temperature. For F-actin labeling, pollen tubes were washed in 50 mM Pipes buffer, then incubated in enzyme solution containing 1% cellulase and 1% pectinase at 37°C for 15 min. Following incubation, pollen tubes were washed in 50 mM Pipes buffer, and then incubated in 1% Triton X-100 at room temperature for 40 min. Then pollen tubes were washed for three times and incubated in 0.2 nM phalloidin-TRITC in PBS (pH 6.9) buffer for 2 h in darkness. The labeling of MTs was performed according to the method of Lazzaro [57]. Pollen tubes were permeabilized for 2 h in 1% Triton X-100/PBS after freeze shattering, then incubated with the monoclonal antibody against ß-tubulin (Sigma, 1∶1000) and FITC- conjugated secondary antibody (Sigma). Thereafter, all the samples were washed and mounted on slides in 50% glycerol and observed under a Zeiss LSM 510 META laser-scanning confocal microscope.

### FM4-64 Staining to Analyze Vesicle Trafficking in the Pollen Tube

FM4-64 was purchased from Sigma. Loading of pollen tubes with FM4-64 dye was generally achieved according to Parton *et al.* [58] with some modification. Samples were supplemented with 3 µM FM4-64 for 10 min, and then washed three times with culture medium. The samples were observed under LSCM. FM4-64 were excited at 514 nm with a 25 mW argon ion laser operated at full power at an intensity of 3%, achieved by means of neutral density filters, with a nearly closed pinhole and the gain adjusted to below a level of 7.00. Images were collected and processes using Adobe Photoshop 7.0.

### Electron Microscopy

Pollen tubes were cultured in the absence and presence of DCB for 3 d, collected, and fixed in 2.5% glutaraldehyde in 100 mM phosphate buffer (pH 7.2) with 2% (w/v) sucrose at 4°C for 2 h. After washing in 100 mM phosphate buffer, the pollen tubes were post-fixed in 2% osmium tetroxide for 2 h, dehydrated in an ethyl alcohol series, and embedded in Spurr’s resin. Sections were cut using an LKB-V ultramicrotome, viewed with a JEM-1230 electron microscope (JEOL Ltd. Tokyo, Japan), and photographed at several different magnifications.

### Localization of Cellulose and Callose

Labeling of cellulose with calcofluor was carried out as described by Lazzaro *et al.* [59]. Briefly, calcofluor (fluorescent brightener 28, Sigma F-3543, UK) was dissolved in distilled water to a final concentration of 10 mg/ml and then centrifuged at 14000 g for 5 min. The supernatant was diluted 1∶10 into an aliquot of pollen tubes in germination medium. After incubated in calcofluor for 45 min in the dark, pollen tubes were examined immediately by fluorescent microscope (Axioskop 40, Zeiss, Germany). The cell wall material (CWM) was isolated and cellulose content was determined according to the method described by Updegraff [60].

Control and treated pollen tubes were collected by gentle centrifugation, fixed with 3% (w/v) paraformaldehyde for 30 min, and stained with 0.05% aniline blue for 2 min. The stained pollen tubes were observed and photographed in a Zeiss Axioskop 40 microscopy (excitation filter BP395-440, chromatic beam splitter FT460, barrier filter LP 470).

### Immunolabeling of Pollen Tubes for Pectins

Immunofluorescent labeling of pollen tubes was carried out as described by Li *et al.* [59]. The monoclonal antibodies JIM5 and JIM7 were generously provided by Prof. K. Roberts (Norwich, U.K.). JIM5 recognizes epitopes of non- or poorly esterified pectin, and JIM7 recognizes epitopes of highly esterified pectin [61].

Pollen tubes were fixed in 3% paraformaldehyde in PME buffer (50 mM PIPES, 0.5 mM MgCl_2_, 1 mM EGTA, pH 6.8) for 30 min at room temperature. After three rinses in PME buffer and phosphate-buffered saline (PBS, pH 7.2), the pollen tubes were incubated with the monoclonal antibodies for 2.5 h at room temperature. Following incubation, the pollen tubes were rinsed three times with PBS (pH 7.2), incubated with sheep anti-rat IgG antiserum linked to fluorescein isothiocyanate (ICN ImmunoBiologicals, Irvine, CA, USA.) for at least 2 h at room temperature, and washed three times with PBS (pH 7.2). The primary antibody was omitted in the control samples. Observations were carried out with a Bio-Rad MRC 600 laser scanning confocal microscope as described above, with excitation at 488 nm and emission at 522 nm.

### Fourier Transform Infrared (FTIR) Analysis

Pollen tubes were collected by gentle centrifugation, rinsed three times with deionized water, and dried at room temperature in a layer on a barium fluoride wafer. The infrared (IR) spectra of the pollen tubes were recorded using a MAGNA 750 FTIR spectrometer (Nicolet Corp., Japan) equipped with a mercury-cadmium-telluride (MCT) detector. Average spectra resulting from 128 scans were evaluated at a resolution of 8 cm^−1^, and spectrum normalization was performed in order to obtain the relative absorbance. The experiment was performed three times with control and DCB-treated pollen tubes.

## Supporting Information

Figure S1 **Effects of DCB on the normally growing pollen tubes of** ***Pinus bungeana.*** Pollen grains were incubated in the normal medium for 3 d, and then DCB was added to a final concentration of 1 µM. Mean values are shown with SD of three experiments.
(TIF)
