# Supplementary figures and images for: 2, 6-dichlorobenzonitrile Causes Multiple Effects on Pollen Tube Growth beyond Altering Cellulose Synthesis in Pinus bungeana Zucc
Source: PLoS One. 2013 Oct 11;8(10):e76660. doi: 10.1371/journal.pone.0076660 (PMC3795706; doi:10.1371/journal.pone.0076660)

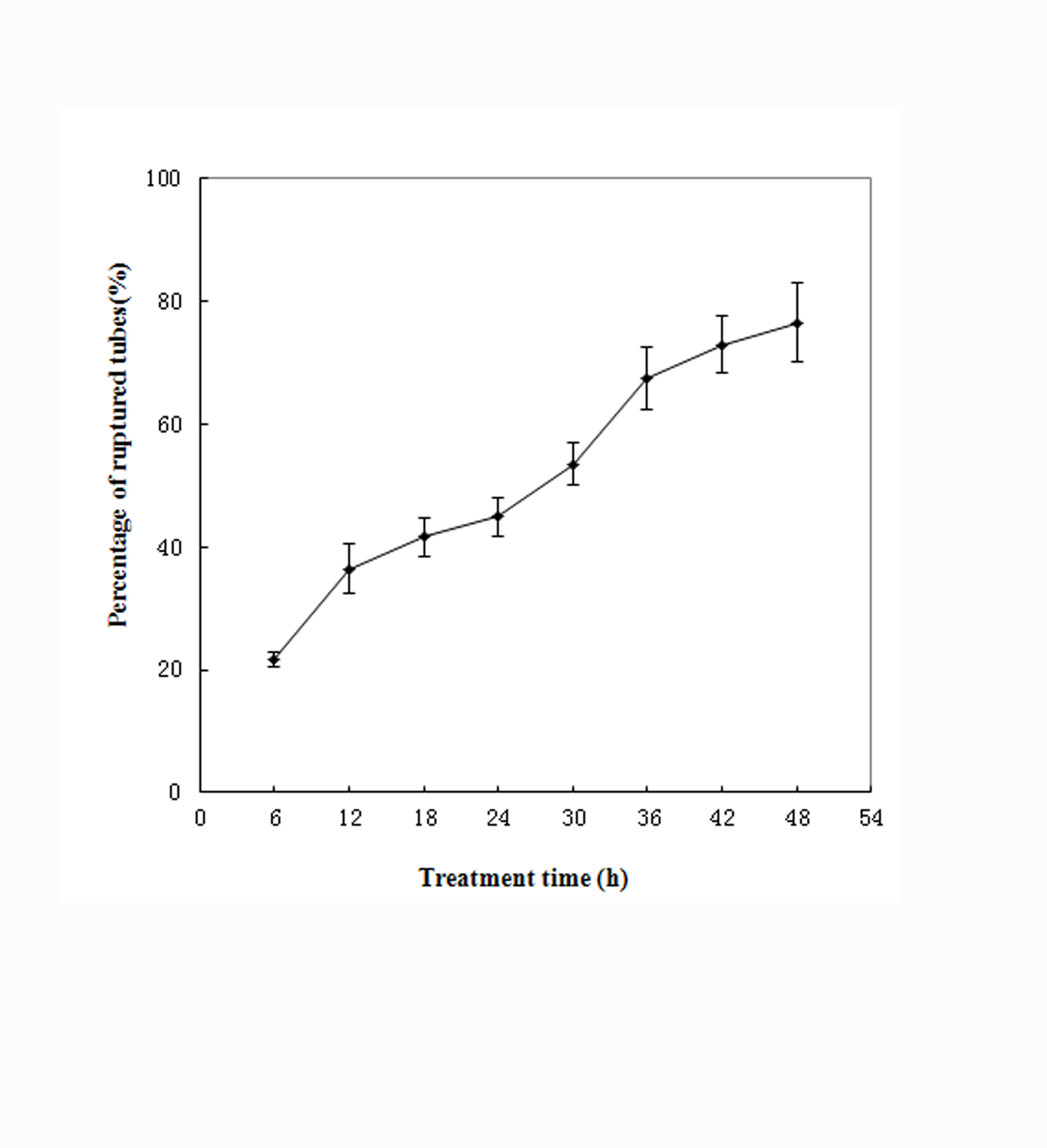

Supplement: Figure S1 — Effects of DCB on the normally growing pollen tubes of Pinus bungeana. Pollen grains were incubated in the normal medium for 3 d, and then DCB was added to a final concentration of 1 µM. Mean values are shown with SD of three experiments. (TIF) [file pone.0076660.s001.tif]
